# Application of crop growth models in crop yield assessment

**DOI:** 10.3389/fpls.2026.1819890

**Published:** 2026-05-11

**Authors:** Zi Ye, Lanqing Liao, Guiqin Qiu, Yurong Xiao, Dajun Huang, Zilin Duan, Shihuang Pu, Jiancheng Wen

**Affiliations:** College of Agronomy and Biotechnology, Rice Research Institute, The Key Laboratory for Crop Production and Smart Agriculture of Yunnan Province, Key Laboratory for Molecular Breeding of Dian-Type Hybrid Japonica Rice, Jingdong County Farmers’ Academician Science and Technology Service Station, Yunnan Agricultural University, Kunming, Yunnan, China

**Keywords:** crop growth model, yield assessment, climate change, yield gaps, adaptation strategies

## Abstract

Global food security is facing formidable challenges due to rising temperatures, frequent extreme weather events, growing water scarcity, cropland reduction, fluctuations in international food trade, and rising food demand. Crop production systems are complex, multi-factor dynamic systems influenced collectively by crop cultivars, climatic conditions, soil properties, and management practices, which exhibit strong spatiotemporal variability. Crop growth models have emerged as essential tools in smart agriculture, integrating knowledge from crop physiology, ecology, meteorology, soil science, and agronomy to simulate crop growth processes dynamically. This systematic review focuses on six key aspects of crop growth modeling: (1) introduction of major crop models; (2) assessing climate change impacts on crop yields; (3) predicting yield potential and yield gaps; (4) identifying yield-limiting factors; (5) formulating adaptation strategies; and (6) challenges and future research directions. Future research should focus on the deep integration of crop growth models with remote sensing, the Internet of Things (IoT), big data, cloud computing, and artificial intelligence technologies to establish intelligent “space-air-ground” decision-making systems that support precision, unmanned, and climate-resilient agriculture.

## 1 Introduction

The report “The State of Food Security and Nutrition in the World (SOFI)” released by the Food and Agriculture Organization of the United Nations (FAO) in 2025 indicates that the global prevalence of undernourishment is projected to decline to 8.2% in 2024 from 8.7% in 2022 (FAO, 2025). Safeguarding global food security remains a critical objective requiring concerted efforts worldwide. Global food security has faced formidable challenges in recent years, driven by a confluence of factors including rising temperature, frequent extreme weather events, growing water scarcity, continuous reduction in grain sown area, fluctuations in international food trade, and rising living standards (Feukam Nzudie et al., 2025; Ribes et al., 2025). Climate change has emerged as one of the most critical threats to agricultural productivity, it directly affects crop growth, phenology, and yield formation (Rezaei et al., 2023; Zheng et al., 2020). These changes not only reduce average yields but also increase inter-annual yield variability, thereby destabilizing food supply chains and exacerbating price volatility. To effectively safeguard food security, it is therefore essential to systematically assess the impacts of climate change on crop production.

Increasing grain production can be achieved either by expanding the cultivated area (Lambin and Meyfroidt, 2011), or by enhancing crop yield on existing farmland (Licker et al., 2010). With growing awareness of ecological systems and an increasing emphasis on harmonious human-nature coexistence, enhancing crop productivity per unit land area, specifically by narrowing the yield gap, has been recognized as a strategic pathway to improve food production while reducing environmental impacts (Pradhan et al., 2015; Ye et al., 2026). Due to pronounced regional differences in climatic conditions, crop yield potential varies considerably across regions. In addition, disparities in the level of production technology further result in large variability in actual yields. Consequently, predicting crop yield potential and quantifying yield gaps are critical for identifying the scope for yield increase and for optimizing the spatial allocation of crop production.

The crop production system is a complex, multi-factor dynamic system jointly influenced by various factors such as crop cultivars, climatic conditions, soil properties, and management practices. These factors exhibit strong spatiotemporal variability, regional heterogeneity, and dependence on managerial experience, consequently introducing substantial uncertainty into regional crop production assessments and presenting challenges for quantification and standardization (Liu et al., 2013; Zhang et al., 2016). Therefore, effectively identifying yield-limiting factors is of great practical significance for formulating appropriate adaptation strategies and unlocking the potential of crop production systems.

As a pivotal tool in smart agriculture, crop growth models are computer-based tools built upon mathematical frameworks and algorithms and through the analysis of extensive experimental data and the synthesis of crop growth and development principles, including their interactions with the environment and management practices (G×E×M). It can rapidly, efficiently, and scientifically quantify the dynamic processes of how climatic conditions, genetic traits, management practices, and soil properties affect crop growth, development, and yield formation (Jones et al., 2003; Keating et al., 2003). Furthermore, they are widely used to assess yield changes due to the impacts of climate change, predicting crop yield potential and yield gaps, identifying yield-limiting factors, and formulating adaptation strategies (Zabel et al., 2021). Also, they have been successfully applied in crop production research spanning from field to global scales (Gao et al., 2025; Jägermeyr et al., 2021), providing a robust technical foundation for systematically evaluating yield potential and identifying yield-limiting factors. Crop growth models, when coupled with climate scenarios (e.g., Historical meteorological data, CMIP5/CMIP6 and RCP/SSP pathways), enable researchers to quantify yield responses under different warming levels, identify vulnerable regions and cropping systems, and evaluate the effectiveness of adaptation strategies—such as adjusted sowing dates, optimized fertilization, supplemental irrigation, and the use of heat-tolerant cultivars (Asseng et al., 2019; Chauhdary et al., 2025; Getachew et al., 2021). By integrating climate, crop, and soil data, these modeling approaches provide robust scientific evidence that supports policymakers and stakeholders in developing proactive, evidence-based adaptation measures, thereby contributing to more resilient and sustainable food systems.

**Figure 1:**
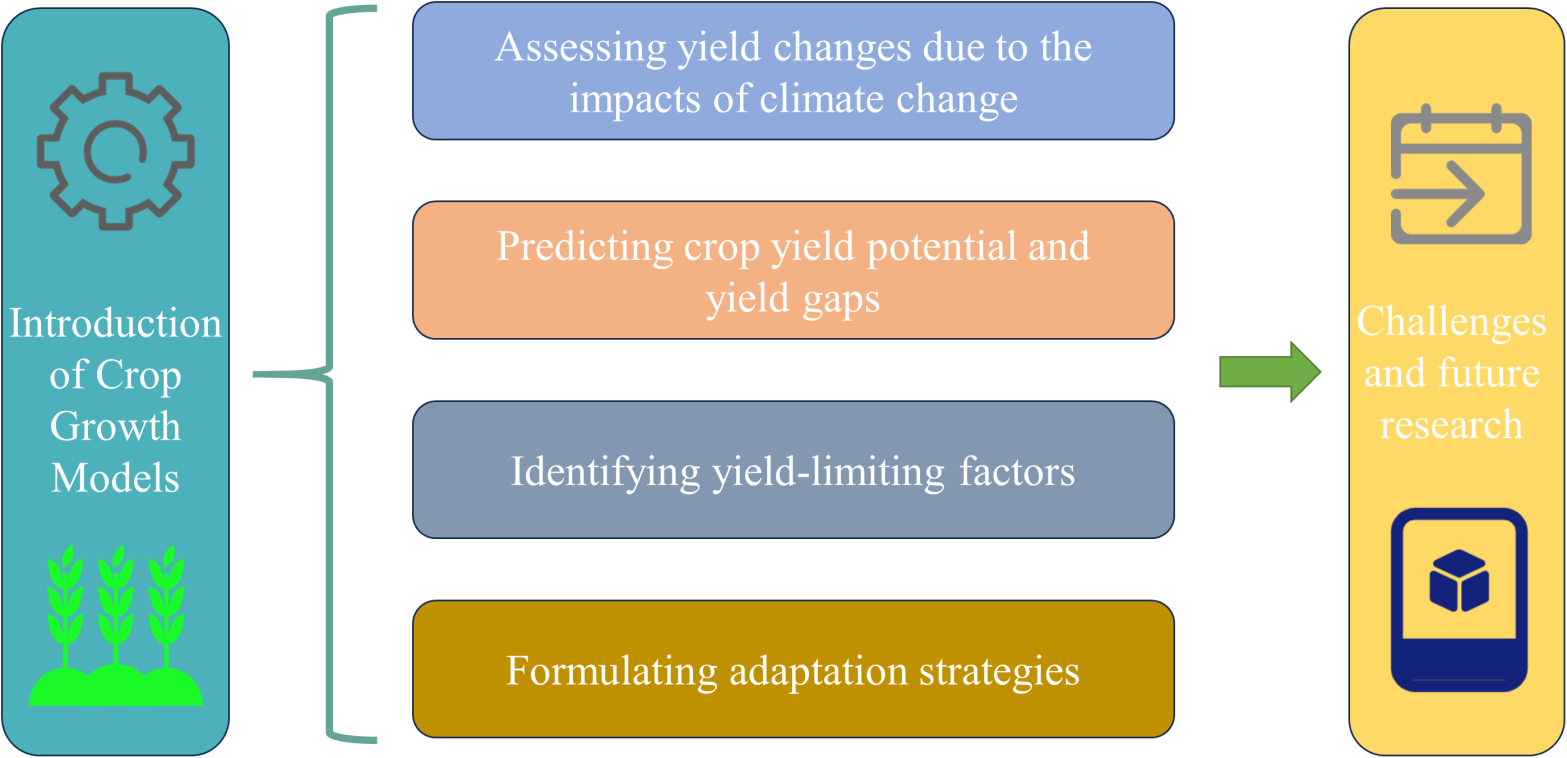
Research framework. This review systematically introduces crop growth models and summarizes their applications in four aspects: assessing yield changes due to the impacts of climate change, predicting crop yield potential and yield gaps, identifying yield-limiting factors, and formulating adaptation strategies, and summarizes the challenges and future research directions for crop growth models.

This systematic review focuses on six key aspects of crop growth modeling (**Figure 1**): (1) introduction of crop growth models; (2) assessing yield changes due to the impacts of climate change; (3) predicting crop yield potential and yield gaps; (4) identifying yield-limiting factors; (5) formulating adaptation strategies; and (6) challenges and future research of crop growth model.

## 2 Introduction of crop growth models

Driven by a progressively deepening understanding of crop growth dynamics and physiological-ecological processes, scientists have employed systems analysis and computer simulation techniques to develop crop growth models by integrating knowledge from crop physiology, ecology, meteorology, soil science, and agronomy. These models can analyze and evaluate crop growth processes and their dynamic interactions with the environment and management practices (Licker et al., 2010; Pradhan et al., 2015; Lobell et al., 2009). Since the 1960s, crop growth models have evolved from simple photosynthesis simulations to comprehensive tools integrating physiological, ecological, soil, and meteorological processes. Currently, dozens of crop models such as DSSAT, APSIM, ORYZA, WOFOST, and AquaCrop have been widely applied in agricultural production research worldwide. To facilitate a clear understanding of the different modeling approaches, the major crop growth models are summarized in **Table 1**, with a focus on four dimensions: affiliation, key features, primary applications, and core modules/algorithms. Because each crop model is developed with different priorities, leading to variations in framework, structure, algorithms, and parameters, every model has its own distinct characteristics.

**Table 1:** Some of internationally recognized crop growth models and their affiliation, key features, primary applications, and core modules/algorithms.

Model name	Affiliation	Key features	Primary applications	Core modules/Algorithms	References
APSIM	CSIRO Agriculture and Food, Australia	Modular, plug-and-play architecture with central engine; generic crop template; parameter-code separation; supports multi-point simulation and species competition.	Farming systems analysis, crop rotations, resource management, climate impact.	Crop, Soil Water, Soil N, Residue, Manager, Erosion, Soil pH.	(Keating et al., 2003)
AFRCWHEAT2	University Copenhagen, Denmark	Mechanistic wheat model emphasizing detailed canopy development via cohort-based leaf and tiller dynamics; integrates water and N stress effects on phenology, leaf expansion, and senescence.	Wheat growth and development, response to water and nitrogen deficits, yield prediction under varying environmental conditions, phenological studies.	Phenology (thermal time, photoperiod, vernalization), Leaf area index (cohort-based), Biomass accumulation, Water and N stress factors, Grain number and yield, Soil water balance, N uptake and partitioning.	(Porter, 1993)
AquaCrop	University of Cordoba, Spain	Water-driven, simple and robust; simulates canopy cover, transpiration, biomass water productivity, harvest index; few parameters.	Water productivity, deficit irrigation, yield response to water, climate change.	Soil water & salt balance, Canopy cover, Transpiration, Biomass water productivity, Harvest index.	(Vanuytrecht et al., 2014)
CropGrow	Nanjing Agricultural University, China	Comprehensive rice/wheat model; includes phenology, organogenesis, photosynthesis, partitioning, yield/quality, nutrients, water, 3D visualization; coupled with GIS/RS.	Regional productivity, climate impact, ideal plant design, digital decision support.	Physiological development time, Organogenesis, RUE, Carbon & N flow, 3D visualization, Model-GIS-RS coupling.	(Yan Zhu et al., 2020)
CropSyst	Washington State University, USA	Multi-year, multi-crop, daily time-step; generic crop parameters; integrated with GIS and weather generator; simulates erosion and salinity.	Cropping systems analysis, water and N budgets, long-term sustainability, watershed simulation.	Water & N budgets, Crop phenology, Biomass, Yield, Erosion, Salinity, GIS linkage.	(Stöckle et al., 2003)
DSSAT	University of Florida, USA	Modular cropping system with 16 crops, unified soil module, CROPGRO template, database tools, decision support.	Agronomic research, yield forecasting, climate change impact, management optimization.	Weather, Soil, Plant (CERES/CROPGRO), SPAM, Management, Pest.	(Jones et al., 2003)
DAISY	Aarhus University, Denmark	Open, component-based 1D soil-crop-atmosphere model; detailed C, N, and water dynamics; supports multiple soil columns and intercropping.	Agroecosystem dynamics, water and N leaching, organic matter turnover, intercrops.	Soil water, Heat, C & N cycling, Crop growth, Organic matter pools, Manager, Open C++ API.	(Abrahamsen and Hansen, 2000)
EPIC	Texas A&M AgriLife Research, USA	Comprehensive, process-based model simulating crop growth, soil erosion, nutrient cycling, and hydrology; integrates multiple management practices; originally designed to assess erosion impacts on productivity.	Soil erosion assessment, crop productivity, climate change impact, water quality, bioenergy crops, nutrient cycling, long-term sustainability, watershed management.	Crop growth (RUE-based), Soil water balance, Nitrogen and phosphorus cycling, Carbon dynamics, Soil erosion (USLE/RUSLE), Hydrology, Management practices (irrigation, tillage, fertilization), CO_2_ effects.	(Jones et al., 1991)
Expert-N	University of Hohenheim, Germany	Modular modeling system with interchangeable sub-models (CERES, SPASS, SUCROS); supports user-defined routines.	Model comparison, structural uncertainty analysis, N dynamics, crop growth processes.	Water flow (Richards), Heat, C & N turnover, Crop growth (multiple), Flexible module exchange.	(Priesack et al., 2006)
GLAM	University of Leeds, UK	Large-area crop model; uses SEMAC for internal consistency; improves drought stress simulation on leaf senescence and biomass partitioning.	Regional climate impact, water stress simulation, large-area yield prediction.	Phenology, LAI, Biomass, Transpiration, Water stress, SEMAC simultaneous equations.	(Droutsas et al., 2019)
HERMES	Institute of Landscape Systems Analysis, Leibniz Centre for Agricultural Landscape Research, Germany	1D agroecosystem model; SUCROS-based generic crop growth; coupled C and N cycles; CO_2_ effects; suitable for climate impact assessment.	Climate change impact, crop rotation, N fertilization recommendation, water & N dynamics.	Soil water (capacity/Richards), Soil C & N, Generic crop growth, CO_2_ response, Management.	(Kersebaum, 2007) (Graß et al., 2015)
InfoCrop	Indian Agricultural Research Institute, India	Generic tropical crop model; integrates pest damage and GHG emissions (CH_4_, N_2_O); simulates soil C and N dynamics; user-friendly.	Tropical agriculture, pest impact, GHG emissions, soil carbon, integrated assessment.	Phenology, Photosynthesis, Partitioning, Pest damage, Soil C & N, GHG emissions, N balance.	(Aggarwal et al., 2006a, Aggarwal et al., 2006b)
LINTUL3	Wageningen University, The Netherlands	Light-driven; uses RUE and LAI; N stress quantified via Nitrogen Nutrition Index (NNI), affecting leaf area, partitioning, and senescence.	Nitrogen-limited growth, potential and water-limited production, light interception analysis.	PAR interception, RUE, Nitrogen Nutrition Index, Leaf area development, N uptake & demand.	(Shibu et al., 2010)
LPJmL	Potsdam Institute for Climate Impact Research, Germany	Global dynamic vegetation model with crop functional types (CFTs); simulates C and water cycles under land use change.	Global biogeochemistry, land use change, carbon & water cycles, crop yield at large scale.	Plant functional types (PFTs), Crop functional types (CFTs), C & H_2_O fluxes, Phenology, Irrigation, Residues.	(Bondeau et al., 2007)
MCWLA	Chinese Academy of Science, China	Large-area process model; coupled photosynthesis-stomatal conductance; Bayesian MCMC for optimization; simulates CO_2_, temperature, VPD effects.	Regional crop productivity, climate variability, CO_2_ fertilization, uncertainty analysis.	Photosynthesis-stomatal conductance, Canopy development, Soil water, Bayesian inversion, MCMC.	(Tao et al., 2009)
MONICA	Institute of Landscape Systems Analysis, Leibniz Centre for Agricultural Landscape Research, Germany	1D agroecosystem model; SUCROS-based generic crop growth; coupled C and N cycles; CO_2_ effects; suitable for climate impact assessment.	Climate change impact, crop rotation, N fertilization recommendation, water & N dynamics.	Soil water (capacity/Richards), Soil C & N, Generic crop growth, CO_2_ response, Management.	(Nendel et al., 2011)
O’Leary-model	Agriculture Victoria Research, Australia	Wheat-specific state-variable model; detailed soil water and N dynamics; simulates grain number and size; validated for semi-arid rotations.	Wheat growth & yield, fallow-wheat rotations, N & water limitations, tillage effects.	Soil water (cascading), Soil N & C, Phenology, Biomass, Grain number & size, Water use efficiency.	(O'Leary et al., 1985)
ORYZA	IRRI, Philippines	Lowland rice-specific; simulates potential, water-limited, and N-limited production; v3 adds soil C, N, root growth, and water-N coupling.	Irrigated & rainfed lowland rice, water-saving technologies, N management, climate change.	Phenology, Canopy photosynthesis, Biomass partitioning, N dynamics, Soil water (PADDY), Water stress.	(Bouman and Laar, 2001)
RZWQM	USDA, USA	1D root-zone water quality model; integrates SHAW energy balance, DSSAT crop modules, pesticide fate; supports tile drainage and auto-calibration.	Water quality, pesticide fate, N leaching, tile drainage, agricultural management.	Richards equation, Macroporosity, C & N pools, Pesticide, DSSAT crop modules, SHAW energy balance, PEST.	(Ma et al., 2001)
SIRIUS	Rothamsted Research, UK	Wheat-specific mechanistic model; emphasizes phenology via leaf number and vernalization; N stress mainly affects leaf area; extended to grain protein fractions.	Wheat development & yield, phenology, protein composition, climate change impact.	Leaf number, Phyllochron, Vernalization, RUE, Canopy development.	(Jamieson et al., 1998)
SALUS	Michigan State University, USA	Sustainable land-use system; integrates CERES crops, Century SOM, time-to-ponding water balance; supports long-term rotations.	Long-term sustainability, tillage & residue effects, cropping systems, land use assessment.	Crop rotations, Tillage, Residue, Water balance (time-to-ponding), Soil C & N (Century-based), Management.	(Basso et al., 2006)
SiriusQuality	INRA, France	Wheat-specific mechanistic model; emphasizes phenology via leaf number and vernalization; N stress mainly affects leaf area; extended to grain protein fractions.	Wheat development & yield, phenology, protein composition, climate change impact.	Leaf number, Phyllochron, Vernalization, RUE, Canopy development, Grain N & protein fractions.	(Martre et al., 2006)
SSM-Wheat	University of Florence, Italy	Simple, mechanistic wheat model; uses biological day, RUE, and modified linear harvest index; transparent and easy to use.	Wheat yield prediction, water & N limitation, genetic analysis, educational purposes.	Biological day, Phenology, LAI (power function), RUE, Modified linear HI, Soil water & N balances.	(Soltani et al., 2013)
STICS	European Food Safety Authority (EFSA), Italy	Generic multi-crop; modular; simulates row crop radiation, detailed management (mulch, organic fertilizer), and ammonia volatilization.	Agronomic diagnosis, environmental impact, crop rotation, management optimization.	Phenology, LAI, RUE, Row crop radiation, Management (mulch, organic fertilizer), N volatilization.	(Brisson et al., 2003)
WOFOST	University of Göttingen, Germany	Light-driven; Gaussian integration for canopy photosynthesis; classic water bucket; widely used for regional yield prediction.	Potential & water-limited yield, regional yield forecasting, climate change impact, land evaluation.	Phenology, Canopy photosynthesis (Gaussian), RUE, Maintenance & growth respiration, Soil water (free drainage).	(de Wit et al., 2020)

The Agricultural Model Intercomparison and Improvement Project (AgMIP), a global collaborative effort involving more than 60 institutions worldwide, has significantly promoted model intercomparison and integration, providing scientific support for climate change impact assessment and food security analysis (AgMIP, 2025). This project’s research has demonstrated that utilizing a multi-model ensemble approach can significantly reduce simulation uncertainties which caused by model structure, model parameters, and model input, especially under climate change conditions (Asseng et al., 2013; Bassu et al., 2014; Chapagain et al., 2022; Martre et al., 2015; Wallach et al., 2020). Although the inherent diversity among models is a major source of structural uncertainty, it also makes multi-model ensembles valuable for capturing a wider range of possible responses and improving the reliability of uncertainty assessments (Chapagain et al., 2022; Wallach et al., 2016). Currently, multi-model ensemble simulation assessment is widely applied in crop yield prediction, climate change impact assessment, and carbon markets (Basso et al., 2025; Ye et al., 2020).

## 3 Assessing yield changes due to the impacts of climate change

**Figure 2:**
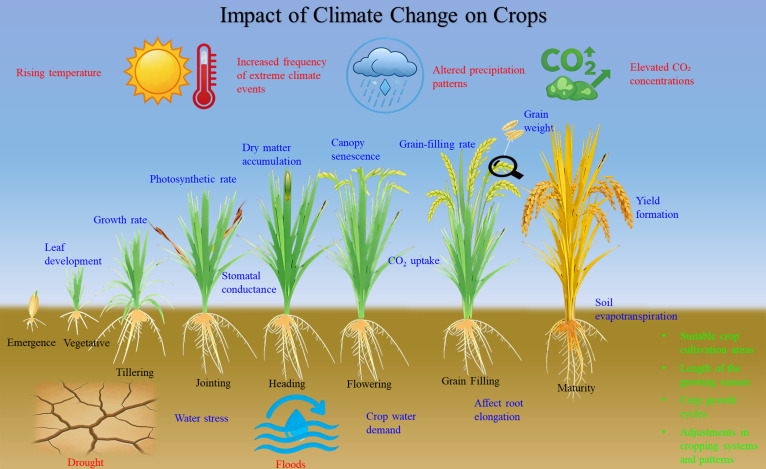
Impact of climate change on crops. The red text in the figure represents the main manifestations of climate change, the black text indicates the crop growth period, the blue text shows the physiological processes affected by climate change, and the green text represents the regional-scale impact of climate change on the crops.

The World Meteorological Organization’s (WMO) “State of the Global Climate 2025” released in March 2026 stated that the Earth’s energy imbalance is highest in the 65-year record, and the period confirms 2015–2025 hottest 11 years on record (WMOW.M.O, 2026). With the progression of global warming, the significance of studying the impacts of climate change has gained broad recognition. Agricultural production is intrinsically linked to climatic resources. Climate change affects crop production through rising temperatures, altered precipitation patterns, increased frequency of extreme climate events, and elevated CO_2_ concentrations (WMOW.M.O, 2026; Zahoor et al., 2023). At the crop physio-biochemical scale, climate change affects key physiological processes including leaf development (White et al., 2012), growth rate (Ottman et al., 2012), photosynthetic rate (Khan et al., 2025), canopy senescence (Kadam et al., 2014), and root elongation (Calleja-Cabrera et al., 2020). Furthermore, elevated temperatures accelerate grain-filling rate, consequently reducing grain weight (Cotrina et al., 2023). However, for winter crops, particularly in regions with low growing-season temperatures, warming can conversely benefit dry matter accumulation and yield formation (Rezaei et al., 2023; Zheng et al., 2020). Furthermore, rising temperatures can increase soil evapotranspiration and crop water demand, potentially leading to water stress. This stress condition often reduces stomatal conductance, thereby limiting CO_2_ uptake and ultimately impairing the accumulation of assimilates (Wang et al., 2022; Zhang et al., 2023). At the regional scale, rising temperatures have facilitated the expansion of suitable crop cultivation areas, extended the length of the growing season, shortened crop growth cycles, and prompted adjustments in cropping systems and patterns (e.g., shifts in crop types and the adoption of multiple cropping (He et al., 2020; Qu et al., 2026). The potential impacts of climate change on crops are summarized in **Figure 2**.

Crop growth models, combined with historical meteorological data and future climate scenarios, can simulate crop growth and development under different climatic conditions. It becomes feasible to assess the impacts of climate change on crop production across regional and broader scales. While, traditional field experiment methods face challenges in revealing the impacts of climate change on crop growth and development at large scales, as their findings are typically confined to specific local conditions. Therefore, crop growth models offer distinct advantages in assessing the impacts of climate change on crop production at large spatial scales and can provide valuable information to support decision-making. For instance, utilizing a multi-model ensemble comprising four wheat growth models (CERES-Wheat, Nwheat, WheatGrow, and APSIM-Wheat), Ye et al. (2020) assessed the impacts of global warming scenarios of 1.5 °C and 2.0 °C on winter wheat production in China, and it indicated that under conditions of elevated temperature coupled with increased CO_2_ concentration, wheat yields in the northern winter wheat region would benefit from a positive effect, whereas the southern region would experience a negative effect. By integrating CMIP6 climate scenarios with the PyAEZ crop model, Upreti et al. (2025) assessed future maize yield trajectories in the Ganges and Mekong River deltas, and revealed the vulnerability of maize production in these regions and provided suitability zoning to inform climate adaptation planning. Using a multi-model ensemble from CMIP6 and the CROPWAT irrigation model to simulate the irrigation water requirements for dry-season maize in Bangladesh under future climate scenarios revealed that rising temperatures and decreasing effective rainfall will lead to a significant increase in both crop water demand and irrigation water requirements for maize (Islam et al., 2025). Chadalavada et al. (2022) used the CERES-sorghum model driven by a multi-model mean of 20 bias-corrected CMIP5 climate projections (RCP 4.5 and 8.5) to assess future climate change impacts on post-rainy season sorghum yields across eight states in India, finding that yields are projected to increase by up to 89% by the end of the century due to higher rainfall and elevated CO_2_ levels, despite rising temperatures. Using machine-learning emulators trained on eight global gridded crop models and 21 CMIP5 climate models, Liu et al. (2021) projects that by 2080 under RCP8.5, climate change will significantly increase interannual wheat yield variability in 18% of global harvested areas (especially hot, low−fertilizer regions) and decrease it in 44%, with temperature change being the dominant driver in 72% of areas. Crop growth models serve as a versatile tool for quantifying climate–crop interactions, identifying regional vulnerabilities, and assessing yield variability, thereby providing essential evidence for climate adaptation planning.

## 4 Predicting crop yield potential and yield gaps

One of effective ways to improve food production is to enhance crop productivity per unit land area, specifically by narrowing the yield gap (Pradhan et al., 2015; Ye et al., 2026). Estimating yield levels and quantifying the yield gap between actual and potential yields help clarify the scope for yield enhancement, which is crucial for ensuring national food security. Yield gap research originated in the 1974s when the International Rice Research Institute (IRRI) first introduced the concept. As research has progressed, the conceptual framework of yield gap analysis has become increasingly comprehensive.

**Figure 3:**
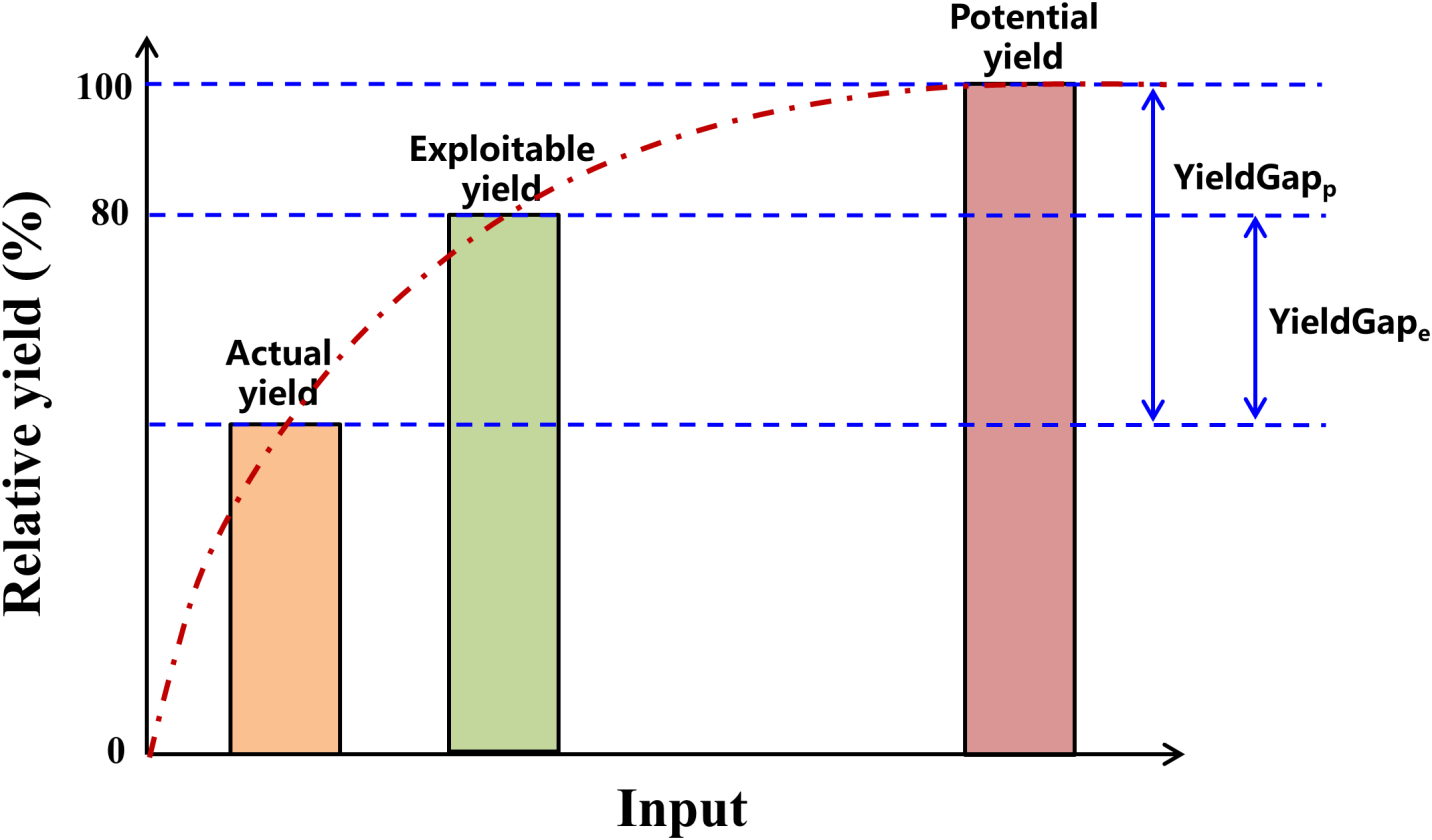
A conceptual framework between actual yield, exploitable yield, potential yield and input. The red dotted line indicates the relationship between yield and input. Two definitions of yield gaps are indicated based on the differences between actual yield and exploitable yield (YieldGap_e_, which is the exploitable yield gap), actual yield and potential yield (YieldGap_p_, which is the theoretical maximum yield gap).

By summarizing the previous research which focus on yield level and yield gaps (Guilpart et al., 2017; Laborte et al., 2012; van Ittersum et al., 2013), this study proposes a novel conceptual framework for yield gaps between actual yield, exploitable yield, potential yield and input (**Figure 3**). Potential yield, also referred to as yield potential, is defined as the maximum theoretical yield achievable by a specific crop cultivar in a given region without limitations from water, nutrients, pests, diseases, or weeds. This yield level represents the uppermost production ceiling for a crop in a specific area and is primarily determined by the local climatic conditions (Bonner, 1962; Evans and Fischer, 1999; Grassini et al., 2009). Exploitable yield refers to the maximum yield actually achieved in experimental plots under optimal agronomic management, without physical, biological, or economic constraints, within a specific temporal and ecological zone (Ittersum and Rabbinge, 1997; Lgabriela et al., 2008). When water and nutrient demands are fully met throughout the growing season, crops can optimally utilize available light and temperature resources, maximize CO_2_ fixation, and efficiently convert it into biomass. Some researchers argue that achieving potential yield through perfect management practices is impractical in actual production systems. This approach typically requires substantial inputs of coast, labor, resources, fertilizers, and pesticides, which not only reduce economic returns but may also lead to significant environmental concerns, such as excessive nutrient loss or eutrophication, biodiversity loss, or heightened greenhouse gas emissions (Maúre et al., 2021). When farmers’ yields reach approximately 75–85% of the potential yield, the rate of yield increase slows with additional inputs, indicating an inflection point. Therefore, 75–85% of the potential yield is defined as the exploitable yield (van Ittersum et al., 2013). Actual yield refers to the quantity of grain actually harvested by farmers in a given region, determined collectively by local climatic conditions, soil properties, cultivar characteristics, and commonly adopted cultivation practices. It represents the real-world yield level of that area. The red dotted line in the **Figure 3** illustrates the yield response to increasing input levels. Initially, yield increases rapidly with additional inputs, followed by a steady growth trend as inputs continue to rise. When the yield level reaches the exploitable yield, the input-output ratio is maximized. Beyond this point, further increases in inputs result in only marginal yield gains until the yield approaches the potential yield level, after which no further increase in yield is observed despite additional inputs. At this stage, the maximum theoretical yield ceiling for the region is achieved. Consequently, YieldGap_e_ is defined as the yield gap between exploitable yield and actual yield, reflecting the relatively achievable high-yield level under current production conditions through the optimization of management practices. YieldGap_p_ is defined as the yield gap between potential yield and actual yield, representing the scope for increasing actual yield toward the maximum theoretical yield.

Predicting crop yield potential and yield gaps serves as a key tool for identifying opportunities for yield enhancement and optimizing the spatial allocation of agricultural production. Therefore, a critical question arises: how can crop yield potential and gaps be assessed in a scientifically robust and efficient manner? Overall, current research predicting yield potential and yield gaps primarily employs approaches such as field experiments, yield contests, farmer surveys, and crop modeling simulations (Lobell et al., 2009; van Ittersum et al., 2013). The first three approaches are relatively straightforward and highly operable, allowing for controlled variable experiments to analyze multiple factors involved in crop production within specific regions (Zhang et al., 2016). However, these methods require substantial data of high quality and quantity, and involve significant investments in labor, material resources, and time. Crop growth models have been demonstrated to be superior to statistical methods for predicting yield potential and yield gaps, as they are time- and cost-efficient, enabling quantification across extensive areas by integrating regional data on climate, soil, and cultivar information (van Ittersum et al., 2013). For example, in assessing maize potential yield and yield gaps in China, Meng et al. (2013) estimated the actual yield from farmer surveys, the attainable yield from 137 high-yielding plots, and the potential yield by integrating the Hybrid-Maize model with documented high records, ultimately revealing substantial yield gaps. Gobbett et al. (2016) demonstrated that closing the yield gap between actual yield and the exploitable yield (defined as 80% of the APSIM-simulated potential yield) could enhance Australia’s total wheat production by 15.3 million tonnes, representing a surge of 72%. An analysis of global yield gaps for ten major crops (1975-2020) by Gerber et al. (2024) showed that while over 60% of maize area saw steady growth, only 12% of rice area did. Furthermore, 84% of rice and 56% of wheat planting areas encountered diminishing opportunities for narrowing yield gaps. Ye et al. (2026) used a multi-model ensemble of four wheat growth models combined with Random Forest analysis to assess wheat yield potential and gaps and stability across China from 1981 to 2020, revealing that northern regions have high potential but low stability, management factors are the dominant drivers, and closing yield gaps could increase national production by 46–57%. Thus, crop growth models provide a powerful and efficient approach for large−scale assessment of yield potential and yield gaps, which is essential for guiding yield improvement and optimizing production distribution.

## 5 Identifying yield-limiting factors

Agricultural production systems are complex, multifactorial dynamic systems whose yield formation is collectively influenced by multiple environmental factors and human management practices. Scientifically identifying yield-limiting factors to crop yield improvement is of great practical significance for optimizing management strategies, coordinating interaction effects among factors, and unlocking the potential of crop production systems. Changes in climatic factors have amplified the instability of crop production systems and pose a potential threat to global food security (Gray et al., 2016; Ribes et al., 2025; Yuan et al., 2023). Cultivar characteristics play a decisive role in crop yield formation. Through the breeding of high-yielding crop varieties, significant yield improvements can be achieved at regional scales (Yuan, 2017). Advances in modern breeding technologies have significantly boosted regional crop productivity through the development and widespread adoption of high-yielding cultivars (Deng et al., 2025; Yuan, 2017). Management practices represent a faster-acting yield regulation approach than breeding. The precise management of external growth factors through timely inputs provides crops with optimized growing conditions, leading to substantial yield improvements. The choice of sowing date critically influences the allocation efficiency of light and temperature resources across the crop developmental phases. Fertilizer application serves as a crucial source of crop nutrients and plays a significant role in yield enhancement (Cassman and Dobermann, 2022; Zhang et al., 2015). Water management plays an indispensable role in crop growth and development, nutrient uptake and translocation, and photosynthetic processes (Calvino and Sadras, 2002). Soil, as the fundamental medium for crop growth, not only supplies essential nutrients but also directly influences crop development and yield formation through its physical and chemical properties (Wood et al., 2018). Soil structure governs the growth and development of crop root systems, which in turn determines the efficiency of water and nutrient uptake by plants. The sustained use of organic fertilizers helps preserve and boost soil fertility, enhances soil physical properties, and ultimately leads to improved crop yields and more efficient nutrient utilization (Huang et al., 2019). However, unsustainable practices such as excessive fertilizer application and flood irrigation can lead to soil nutrient leaching and compaction, which adversely affect root development and nutrient uptake (Gu et al., 2015), ultimately resulting in yield reduction (Maúre et al., 2021).

To conclude, crop yield is jointly influenced by multiple factors including climate, genetic potential, agronomic practices, and soil properties (Asseng et al., 2011; Maúre et al., 2021; Yuan, 2017). Crop growth models, recognized for their mechanistic, dynamic, and predictive capabilities, enable the decomposition of individual factor contributions to final yield (Jones et al., 2003; Keating et al., 2003; Sumberg, 2012; van Ittersum et al., 2013). The relative importance derived from these models was used to identify limiting factors for crop yields. For example, Webber et al. (2018) employs a multi-model ensemble of crop and climate models under RCP scenarios to project future yields in Europe, revealing that while climate change will intensify drought stress and reduce rainfed maize yields, winter wheat yields will remain stable; however, elevated CO_2_ offers no benefit during low-yielding years where drought is the dominant driver of losses. Using the APSIM-Oryza model to simulate the impacts of climate change, soil improvement, cultivar renewal, and optimized management practices on rice yield in the Taihu Lake region during the 1980s and 2000s, the results showed a 46.3% increase in rice yield between the two periods, with the four factors contributing -19.5%, 12.7%, 21.7%, and 34.6% to this yield enhancement, respectively (Liu et al., 2013). Using the CERES-Wheat model, Zhang et al. (2013) simulated the impacts of climate, cultivar, and fertilization on yield variability at the Luancheng site in the North China Plain from 1979 to 2012. Their results indicated that climate change caused wheat yield variations ranging from -39% to 20%, while cultivar renewal and fertilizer application contributed 52% and 6.8%, respectively, to the yield increase compared to the 1980s baseline. Therefore, crop growth models serve as a powerful tool for disentangling the individual contributions of climate, genetics, management, and soil factors to crop yield, thereby enabling the robust identification of yield-limiting factors and providing a scientific basis for targeted agronomic interventions.

## 6 Formulating adaptation strategies

**Figure 4:**
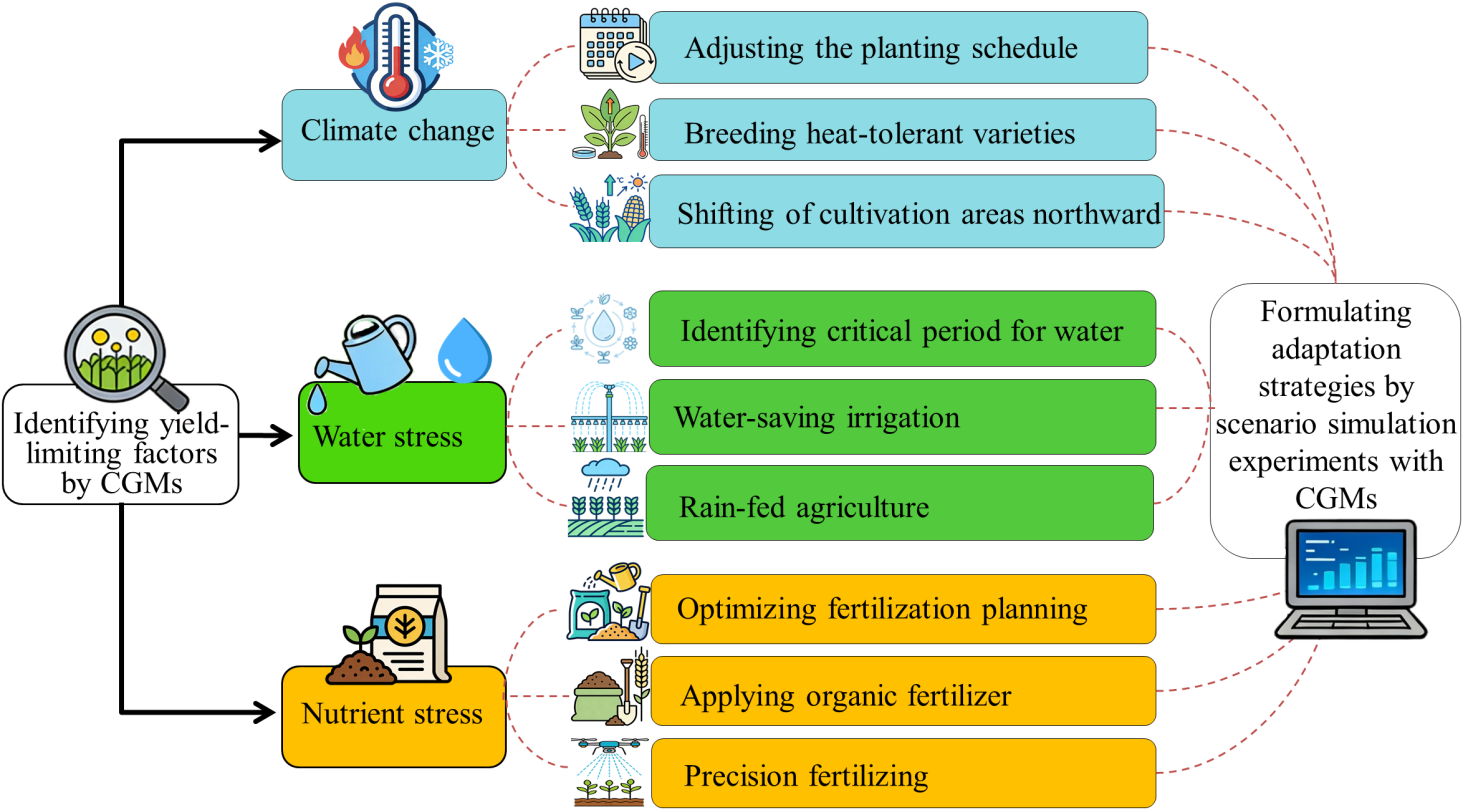
Identifying the yield-limiting factors and formulating adaptation strategies by scenario simulation experiments with Crop Growth Models (CGMs). This article primarily reviews yield-limiting factors—including climate change, water stress, and nutrient stress—that can be accurately identified by crop models, and formulates corresponding adaptation strategies.

Crop growth models enable scenario simulations of crop development, growth, and yield formation under different management practices, making them widely used in research on formulating adaptation strategies to mitigate climate change impacts. Adaptation measures such as adjusting sowing dates, breeding heat-tolerant cultivars, and optimizing irrigation and fertilization have proven effective in counteracting the adverse effects of climate change on crop yields (Challinor et al., 2007; Gouache et al., 2012; Jingsong et al., 2012; Tao et al., 2012). Importantly, region-specific strategies should be developed based on local climatic conditions and production systems. Below, we synthesize recent advances across three key adaptation strategies informed by crop modeling studies (**Figure 4**).

### 6.1 Optimizing sowing dates and cultivar selection

Adjusting sowing dates and adopting climate-resilient cultivars are among the most effective adaptation measures. For example, in the North China Plain, combining high-temperature-tolerant maize cultivars with appropriate sowing date adjustments could increase maize yields by 1.0–15.2% (Tao and Zhang, 2010). A multi-model ensemble study for Mexico demonstrated that updating wheat cultivars with individual or combined genetic traits consistently improved yields under climate change, particularly under rainfed conditions, with greater benefits observed under scenarios of delayed flowering and increased nitrogen application (Hernandez-Ochoa et al., 2019). At the global scale, virtual variety introduction experiments for maize, rice, soybean, and wheat under four warming scenarios revealed that introducing adapted cultivars could offset climate change-induced yield losses in 85% (low warming) and 61% (high warming) of current cultivation areas based on existing varieties (Zabel et al., 2021).

### 6.2 Optimizing irrigation and water management

Water management is critical in drought-prone regions. Using the APSIM-Maize model in Texas, USA, drought-tolerant varieties increased yield by 21% compared to conventional varieties when irrigation was reduced to 50% of baseline levels. Conversely, when the target yield was set at 50% of baseline, drought-tolerant varieties achieved this with 15% less irrigation, demonstrating enhanced water use efficiency (Su et al., 2022). A study across six crops in Australia using the APSIM model from 1910 to 2020 found that while global warming significantly advanced flowering and reduced yields (by 24% under dryland conditions), irrigation alleviated water stress, delayed flowering, broadened the optimal flowering window, and increased average yields by 44%—though it could not fully offset long-term yield penalties caused by rising temperatures (Muleke et al., 2022). Using the DSSAT-CSM-CERES-Sorghum model in Ethiopia, full irrigation combined with early planting effectively mitigated climate-induced yield declines of up to 2 t·ha^-^¹ caused by intensified drought stress (Getachew et al., 2021).

### 6.3 Optimizing nitrogen fertilization

Crop models have been instrumental in designing efficient nitrogen management strategies. Using the Azodyn model for wheat nitrogen management, rational optimization of fertilizer timing reduced nitrogen application by 50 kg·ha^-^¹ while maintaining current yield levels and enhancing grain protein content to exceed 11.5% (Ravier et al., 2021). Similarly, the CERES-Maize model revealed that nitrogen application aligned with crop demand at different growth stages improved nitrogen use efficiency by 8–71% at the farm level and 1–38% at the regional level in China (Wang et al., 2021). By integrating the AZODYN and AFISOL models for pea-wheat intercropping systems in Europe, model-based approaches assisted farmers in designing nitrogen management strategies to achieve high yields (Malagoli et al., 2020). Using the APSIM model on farmer fields of wheat and maize, the findings underscored the effects of climate change on wheat-maize cropping systems and the importance of implementing optimized fertilization and adjusting plant density to mitigate adverse effects (Chauhdary et al., 2025).

Furthermore, crop models also enable the design of ideal crop types for future climates. Senapati et al. (2019) employed the Sirius model combined with RCP8.5 climate scenario data to design ideal wheat ideotypes for high-yield potential in the UK and New Zealand under 2050 climate conditions, achieving yield increases of 43–51% under rainfed conditions and 51–62% under full irrigation. Synthesizing existing research, the development of improved cultivars and the adjustment of agronomic practices can not only counteract the adverse effects of climate change but also capitalize on potential opportunities to enhance crop productivity (Asseng et al., 2019; Hernandez-Ochoa et al., 2019). Collectively, these studies demonstrate that crop growth models serve as an indispensable platform for designing, evaluating, and optimizing region-specific adaptation strategies, encompassing sowing dates, cultivars, fertilization, irrigation, and integrated management, to mitigate climate change impacts, enhance resource use efficiency, and ensure sustainable crop productivity.

## 7 Challenges and future research

The integration of machine learning and deep learning techniques with crop growth models to improve intelligent decision-making has become a major research focus. To date, numerous studies have been carried out in this area. For instance, building upon the agricultural systems modeling approach, Xiao et al. (2024) integrated four machine learning and two deep learning algorithms with a multi-objective genetic algorithm to optimize fertilization, irrigation, and straw incorporation practices. This integrated methodology enabled the design of optimal management prescriptions for the summer maize and winter wheat system in the North China Plain, achieving the triple objectives of stable yield, enhanced carbon sequestration, and reduced greenhouse gas emissions under both historical and future climate scenarios. Xiao et al. (2022) employed three machine learning algorithms, Multivariate Adaptive Regression Splines (MARS), Random Forest (RF), and Boosted Regression Trees (BRT), to predict yields simulated by the APSIM model. Their results demonstrated that precipitation was the dominant contributor to both wheat and maize yields at the regional scale, whereas nitrogen fertilization and irrigation emerged as critical factors influencing productivity at the site-specific scale. A study employing polynomial regression, Long Short-Term Memory (LSTM) networks, and crop modeling revealed that a 1°C temperature increase would lead to global maize yield losses of 6.88%, 4.86%, and 5.61%, respectively. Furthermore, a 10% increase in precipitation could mitigate these temperature-induced losses by 3.98%, 1.05%, and 3.10%, respectively. Under constant temperature conditions, a 10% precipitation increase alone would enhance global maize yields by 0.23%, 1.43%, and 3.09%, respectively (Yin et al., 2022). Feng et al. (2019) combined the APSIM model with a random forest (RF) model to create a hybrid approach, which enhanced the accuracy of crop model simulations in response to extreme climate events. Future research directions should include the development of hybrid models that couple process-based crop growth models with deep learning algorithms, the optimization of multiple management practices to support climate-smart agriculture, improvements in the simulation of extreme climate events, the enablement of spatiotemporal scaling and transfer learning, the enhancement of model interpretability, and the advancement of intelligent decision-making capabilities.

Integrating crop growth models with intelligent sprinkler and drip irrigation systems offers a promising pathway to conserve water resources and enhance water productivity (Li et al., 2019). For instance, by combining a reinforcement learning algorithm with the DSSAT model, Chen et al. (2023) developed a precision irrigation model that provides optimal irrigation strategies to maximize cotton yield while reducing water consumption, aligning with the goals of sustainable water management and climate adaptation. However, a major challenge remains in improving the simulation accuracy for multiple stressors, as current crop growth models are predominantly developed and parameterized for single stressors, leaving the complex interactions among multiple stress factors largely underexplored. Although a conceptual framework for multi-stress response has been proposed (Webber et al., 2022), practical algorithms that reliably account for actual multi-stress interactions require further development. Therefore, future research should focus on advancing intelligent irrigation systems coupled with crop models to improve water productivity, while also refining multi-stress algorithms to better capture the synergistic and antagonistic effects of combined abiotic stresses on crop growth and yield. Moreover, cultivar parameters in crop models are difficult to link directly with underlying genes or QTLs and cannot be obtained through rapid phenotyping. To satisfy the requirements of G×E×M simulations for breeding, interdisciplinary collaboration and resource investment are needed to develop new models. A three-stage framework has been proposed (Wang et al., 2019): improving key physiological processes, identifying genes/QTLs for model input parameters, and collecting data to quantify polygenic interactions. Combined with this framework offers a feasible pathway for developing next-generation models to support crop improvement.

**Figure 5:**
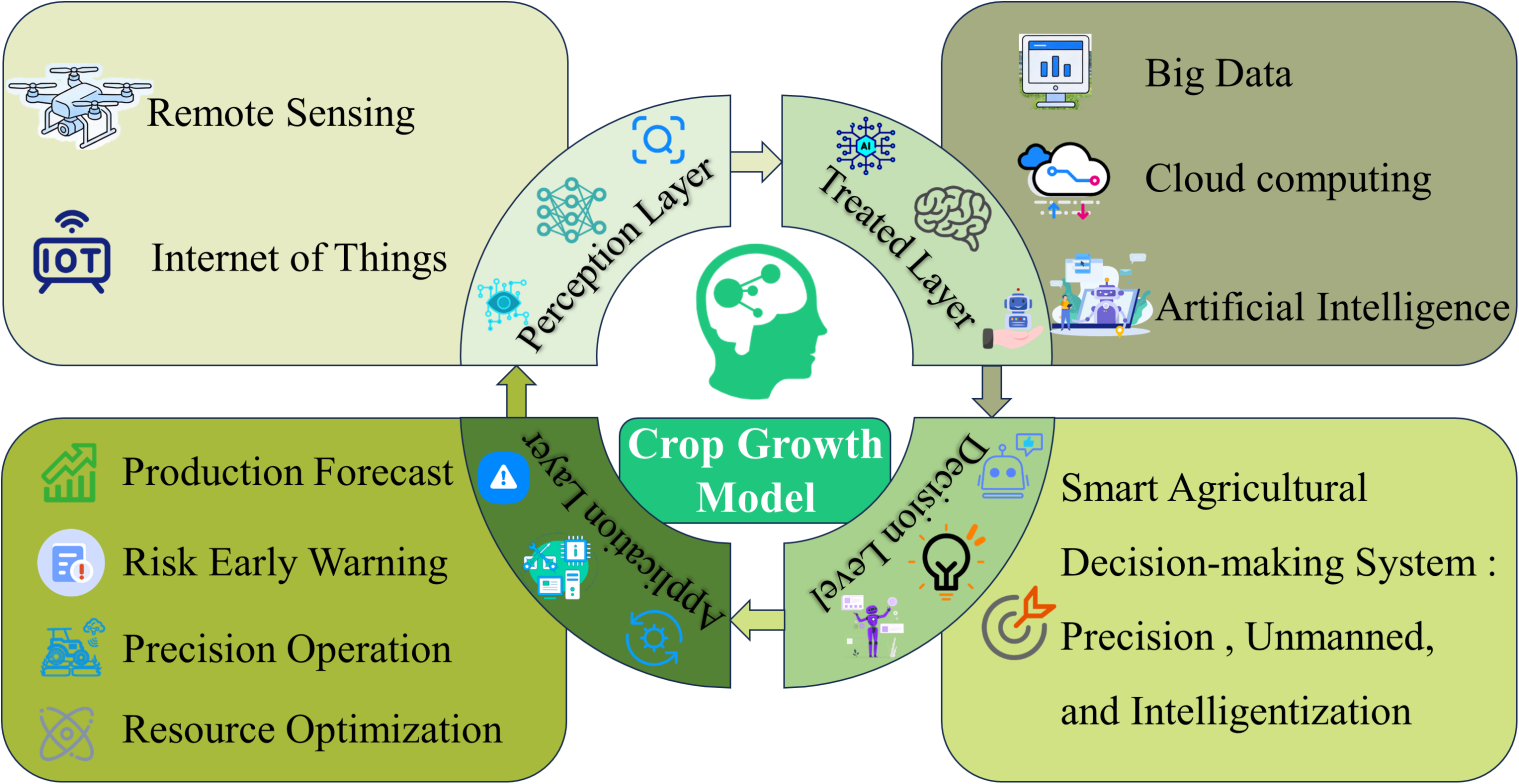
Future research and development directions for crop growth models. Future research should focus on the deep integration of crop growth models with remote sensing, the Internet of Things (IoT), big data, cloud computing, and artificial intelligence technologies to establish an integrated “space-air-ground” intelligent agricultural decision-making system.

In conclusion, future research should focus on the deep integration of crop growth models with remote sensing, the Internet of Things (IoT), big data, cloud computing, and artificial intelligence technologies to establish an integrated “space-air-ground” intelligent agricultural decision-making system (**Figure 5**). This system will enable accurate production forecasting, early warning of climate and pest risks, precision operation, and optimal resource allocation. Ultimately, these advances will drive agriculture toward precision, unmanned management, and full intelligentization, ensuring sustainable and climate-resilient crop production.

## 8 Conclusion

With the continuous advancement of agricultural technology, crop growth models are playing an increasingly vital role in the agricultural sector. As a core technology in smart agriculture, crop growth models have demonstrated great utility in risk assessment, predicting crop yield potential and gaps, identifying yield-limiting factors, and formulating adaptation strategies. With the improvement of data acquisition capabilities and the advancement of model algorithms, crop growth models will play an increasingly important role in global food security assessment, agricultural climate risk management, and cropping system optimization. In the future, crop growth models will be deeply integrated with remote sensing, the Internet of Things (IoT), big data, cloud computing, and artificial intelligence technologies, driving agricultural production toward scientific, intelligent, and unmanned operations.
